# Sociodemographic and behavioral factors associated with controlled hypertension after 9 years of observation of a PURE Poland cohort study

**DOI:** 10.3389/fpubh.2023.1167515

**Published:** 2023-04-21

**Authors:** Katarzyna Zatońska, Alicja Basiak-Rasała, Katarzyna Połtyn-Zaradna, Dagmara Gaweł-Dąbrowska, Maria Wołyniec, Maciej Karczewski, Andrzej Szuba

**Affiliations:** ^1^Division of Population Studies and Prevention of Non-Communicable Diseases, Department of Population Health, Wrocław Medical University, Wrocław, Poland; ^2^Division of Public Health, Department of Population Health, Wrocław Medical University, Wrocław, Poland; ^3^Department of Applied Mathematics, Faculty of Environmental Engineering and Geodesy, Wrocław University of Environmental and Life Sciences, Wrocław, Poland; ^4^Department of Angiology, Hypertension and Diabetology, Wroclaw Medical University, Wrocław, Poland

**Keywords:** hypertension, Poland, cohort, hypertension prevention and control, blood pressure-prevention and control

## Abstract

**Introduction:**

Despite some improvement in awareness and treatment of hypertension, blood pressure control is still below expectations in Poland. The aim of the study was to analyze the secular trend of hypertension prevalence in the PURE Poland cohort study over 9 years of observation and to analyze factors associated with controlled HT.

**Methods:**

The study group consisted of 1,598 participants enrolled in a Prospective Urban and Rural Epidemiological Study (PURE), who participated both in baseline (2007–2010) and 9-year follow-up (2016–2019). Hypertension was ascertained on the basis of (1) self-reported hypertension previously diagnosed by the physician, (2) self-reported anti-hypertensive medication, and/or (3) an average of two blood pressure measurements ≥140 mmHg systolic BP and/or ≥90 mmHg diastolic BP.

**Results:**

The prevalence of hypertension increased from 69.4% at baseline to 85.9% at 9-year follow-up. The chance of HT was 8.6-fold higher in the oldest vs. the youngest age group [OR 8.55; CI 4.47–16.1]. Male sex increased the chance for hypertension over 3-fold [OR 3.23; CI 2.26–4.73]. Obesity, according to BMI, increased the chance of HT 8-fold [OR 8.01; CI 5.20–12.8] in comparison with normal body weight. Male sex decreased the chance of controlled HT after 9 years [OR 0.68; CI 0.50–0.92]. There was no statistically significant association between controlled HT and age or place of residence. Higher and secondary education increased the chance of controlled HT over 2-fold in comparison with primary education [OR 2.35; CI 1.27–4.34, OR 2.34; CI 1.33–4.11]. Obesity significantly decreased the chance of controlled HT after 9 years in comparison with normal body weight [OR 0.54; CI 0.35–0.83].

**Conclusion:**

Factors significantly increasing the chance for controlled hypertension after 9 years were female sex, secondary and tertiary education, normal body weight, and avoiding alcohol drinking. Changes in lifestyle, with special emphasis on maintaining normal body weight, should be the basis of prevention and control of HT.

## 1. Introduction

According to the latest estimates, arterial hypertension (HT) affects 1.28 billion people worldwide—one in four men and one in five women are hypertensive (1). About half of the affected people are unaware of their condition. The prevalence of HT differs depending on the economic status of the country. It is estimated that two-third of HT patients live in countries with low or medium socio-economic status. HT is considered to be the main factor responsible for premature mortality. It affects the functioning of the whole cardiovascular system and other organs, e.g., kidneys and brain. HT risk factors can be divided into modifiable and non-modifiable. Non-modifiable risk factors include a family history of HT, age of over 65 years, and co-existing diseases such as diabetes or kidney disease. Modifiable risk factors include an unhealthy diet (excessive salt consumption (2), a diet high in saturated fat and trans fats, and low intake of fruits and vegetables), low physical inactivity, consumption of alcohol, and being overweight or obese (1). In 2019, the global prevalence of HT was 34% for men and 32% for women (3). On the contrary, the global analysis of HT prevalence included in the PURE study, which comprised countries from different socio-economic backgrounds, indicated that 40.8% of participants had elevated BP (4). There are still a lot of inequalities in BP prevalence and control between high- and low-income countries. In most high-income countries, HT prevalence decreased over time, whereas in low-income countries the opposite happened. In 2019, HT prevalence was the highest in central and eastern Europe, central Asia, Oceania, southern Africa, Latin America, and the Caribbean (3).

In Poland, according to the NATPOL survey in 2002, 29% of men and women aged 18 and older suffered from HT while 29% of survey participants had high normal blood pressure (5). The results of the WOBASZ study (2003–2005) and the NATPOL 2011 study indicated a high prevalence of HT as a high level of global risk within the population of adults in Poland (6, 7). NATPOL 2011 survey revealed a high prevalence of HT of 32%, and only in ~26% of all hypertensive patient's blood pressure was well-controlled (7). In another Polish survey, the PONS study revealed even higher HT prevalence of 67% in the Polish population in Kielce province (8). The Prospective Urban Rural Epidemiology (PURE) baseline study in Poland which was conducted between 2007 and 2010 showed that 60.28% of the studied population had HT. It was more prevalent among men than women (73.85% vs. 52.22%) (9). A recent nationwide cross-sectional study, LIPIDOGRAM 2015, showed that 49% of participants were hypertensive without significant differences between urban and rural settings (10). Analysis of cross-sectional data of the WOBASZ I and WOBASZ II study (2013–2016) revealed a 12% in HT prevalence between the first and second round of the study (11). Despite some improvement in treatment and BP control, they are still below expectations in the Polish population (12).

Studies on the prevalence of HT, lifetime trends, and BP control in Polish population are limited. The aim of the study was to analyze the secular trend of HT prevalence in the PURE Poland cohort study over 9 years of observation and to analyze factors associated with controlled HT.

## 2. Methods

### 2.1. Settings and participants

The study group consists of participants enrolled in the global Prospective Urban and Rural Epidemiological (PURE) study. The Polish cohort was established between 2007 and 2010, recruiting both urban and rural inhabitants from the Lower Silesia region in Poland. All participants were examined in accordance with the global PURE study protocol (13), which included a questionnaire study [individual health, household, family, food frequency questionnaire, and international physical activity questionnaire (IPAQ)], anthropometric measurements, blood pressure measurement, blood draw, ECG, and spirometry. The recruitment process of the Polish cohort and characteristics of the study group at the baseline were described by Zatońska et al. (14). The baseline cohort consisted of 2,036 adult participants (1,282 women and 754 men), aged 30–85 years (mean age: 54 years, SD ± 10). Participants have been repeatedly invited to the study center every 3 years and were examined with a consistent protocol. The study reports the results of 1,598 participants who took part in both the baseline study and 9-year follow-up (2016–2019).

### 2.2. Hypertension criteria

Hypertension was ascertained on the basis of (1) self-reported hypertension previously diagnosed by the physician, (2) self-reported anti-hypertensive medication, and/or (3) an average of two blood pressure measurements ≥140 mmHg systolic BP and/or ≥90 mmHg diastolic BP (9). Blood pressure measurements were carried out with an automated oscillometric device (Omron Corporation, Tokyo, Japan). The appropriate cuff size has been selected. Participants were advised to sit and rest for 5 min before consecutive blood pressure measurements. The same methodology was applied at the baseline and at 9-year follow-up. Hypertension was considered controlled after 9 years of observation if objectives of the ESH/ESC guidelines were met (BP < 140/90 mmHg).

### 2.3. Other variables

The following variables have been included in the analysis: sex, age, place of residence, education, marital status, attitudes toward tobacco smoking and alcohol consumption, body mass index (BMI), waist-to-hip ratio (WHR), and waist-to-height ratio (WHtR). Education was ascertained based on self-reported highest completed level, e.g., primary, vocational, secondary, and college/university.

The body mass of the participants was measured with the use of the Tanita Ironman Body Composition Monitor Model BC-554 with an accuracy of 0.1 kg. BMI was calculated as weight (kg) divided by height (m) squared. Participants were ascribed to four BMI categories according to the WHO guidelines: underweight (BMI < 18.5 kg/m2), normal weight (BMI 18.5–24.9 kg/m2), overweight (BMI 25.0–29.9 kg/m2), and obesity (BMI ≥ 30.0 kg/m2). The occurrence of abdominal obesity was assessed with the use of waist-to-hip ratio (WHR). Abdominal obesity was determined when WHR in men was ≥0.94 and in women was ≥0.80. Waist-to-height ratio (WHtR) has been assessed as waist circumference divided by body height expressed in the same units. Attitude toward tobacco smoking and alcohol consumption was self-reported by the participants. Participants could have chosen one of three possible answers regarding tobacco consumption: “formerly used tobacco products,” “currently use tobacco products,” and “never used tobacco products.” Regarding alcohol consumption, participants could have chosen between “formerly used alcohol products,” “currently use alcohol products,” and “never used alcohol products.”

### 2.4. Statistical methods

The normality of data was tested using the Shapiro–Wilk test and the assessment of the Q-Q plot. The significance of demographic and clinical data in the control and HT groups was tested using the chi square or Fisher's exact test depending on the number of expected observations. The relationship between 9-year controlled HT and BMI, WHR, and waist-to-height ratio was identified using Fisher's exact test. Analysis of chance for controlled HT after 9 years was performed using generalized linear mixed model analysis with patient ID as a random effect. Effect size is presented as odds ratio with 95% confidence intervals. The presented model is a full model adjusted for the following covariates: sex, age, residence, education, BMI, WHR, tobacco use, alcohol use, and blood pressure medications. Analysis was performed in R for Windows (version 4.0.3) (R Core Team, 2020). Differences were considered statistically significant if the *p*-value was lower than 0.05.

### 2.5. Ethics

The study has been reviewed and accepted by the Bioethics Committee of the Wrocław Medical University and has therefore been performed in accordance with the ethical standards laid down in an appropriate version of the 1964 Declaration of Helsinki (Positive opinion of The Bioethics Committee of the Wrocław Medical University nr KB- 443/2006).

## 3. Results

### 3.1. Prevalence of HT at the baseline

Baseline characteristics of the population considering the prevalence of HT and corresponding risk factors are presented in Table 1. At the baseline, HT was observed in 69.4% of participants. A total of 34.9% of the baseline population was not diagnosed with HT previously; however, BP measurement indicated HT. The occurrence of HT was significantly associated with age, sex, level of education, marital status, BMI, WHR, waist-to-height ratio, and attitude toward tobacco smoking. There was a gradual increase in HT prevalence along with age. HT was the most prevalent in the oldest age group >64 years of age (86.3%) in comparison with 42.8% in the youngest age group <45 years of age. The risk of HT was 8.7-fold higher in the oldest vs. the youngest age group [OR 8.73; CI 5.63–13.9].

**Table 1:** Prevalence of hypertension at baseline and 9-year follow-up with corresponding sociodemographic factors.

	**Baseline % (** ***n*** **)**					**9-year follow-up % (** ***n*** **)**			
**Variable**	**Categories**	**Normal BP**	**Hypertension**	***p*** **-value**	**OR (95% CI)**	**Normal BP**	**Hypertension**	***p*** **-value**	**OR (95% CI)**
Age	Total	489	1,109	-		226	1,372	-	
	<45	57.19 (159)	42.81 (119)	<0.001	-	37.25 (19)	62.75 (32)	<0.001	-
	45–64	27.51 (301)	72.49 (793)		3.54^*^ (2.70–4.65)	19.95 (158)	80.05 (634)		2.38^*^ (1.29–4,28)
	>64	13.72 (31)	86.28 (195)		8.73^*^ (5.63–13.9)	6.49 (49)	93.51 (706)		8.55^*^ (4.47–16.1)
Sex	Women	36.70 (378)	63.30 (652)	<0.001	-	18.35 (189)	81.65 (841)	<0.001	-
	Men	19.89 (113)	80.11 (455)		2.31^*^ (1.82–2.96)	6.51 (37)	93.49 (531)		3.23^*^ (2.26–4.73)
Residence	Urban	31.82 (309)	68.18 (662)	0.26	0.87 (0.7–1.09)	16.68 (162)	83.32 (809)	<0.001	0.57^*^ (0.41–0.77)
	Rural	29.03 (182)	70.97 (445)		-	10.21 (64)	89.79 (563)		-
Education	Primary	18.26 (40)	81.74 (179)	<0.001	-	4.57 (10)	95.43 (209)	<0.001	-
	Trade	32.92 (79)	67.08 (161)		0.46^*^ (0.3–0.71)	10.00 (24)	90.00 (216)		0.43^*^ (0.19–0.9)
	Secondary	28.91 (185)	71.09 (455)		0.55^*^ (0.37–0.81)	13.44 (86)	86.56 (554)		0.31^*^ (0,15–0.58)
	University	37.47 (187)	62.53 (312)		0.37^*^ (0.25–0.54)	21.24 (106)	78.76 (393)		0.18^*^ (0.09–0.33)
Marital status	Married	31.26 (376)	68.74 (827)	0.025	-	14.30 (172)	85.70 (1,031)	0.015	-
	Divorced	35.04 (41)	64.96 (76)		0.84 (0.56–1.26)	21.37 (25)	78.63 (92)		0.61^*^ (0.39–1.0)
	Never married	34.95 (36)	65.05 (67)		0.84 (0.55–1.29)	13.59 (14)	86.41 (89)		1.06 (0.61–1.98)
	Widowed	21.26 (37)	78.74 (137)		1,67^*^(1,15–2,48)	8.05 (14)	91.95 (160)		1.91^*^ (1.12–3.52)
BMI^*^	Normal	53.42 (250)	46.58 (218)	<0.001	-	35.92 (125)	64.08 (223)	<0.001	-
	Overweight	28.13 (182)	71.87 (465)		2.93^*^ (2.29–3.77)	14.48 (74)	85.52 (437)		3.31^*^ (2.39–4.62)
	Obesity	11.80 (57)	88.20 (426)		8.57^*^ (6.20–12.0)	6.54 (27)	93.46 (386)		8.01^*^ (5.20–12.8)
WHR	Abd. obesity	22.03 (226)	77.97 (800)	<0.001	-	13.52 (119)	86.48 (761)	<0.001	-
	Normal	45.69 (260)	54.31 (309)		0.34^*^ (0.27–0.42)	27.37 (107)	72.63 (284)		0.42^*^ (0.31–0.56)
WHR women	Abd. obesity	27.31 (189)	72.69 (503)	<0.001	-	17.16 (105)	82.84 (507)	<0.001	-
	Normal	54.93 (184)	45.07 (151)		-	38.71 (84)	61.29 (133)		-
WHR men	Abd. obesity	11.08 (37)	88.92 (297)	<0.001	-	5.22 (14)	94.78 (254)	<0.001	-
	Normal	32.48 (76)	67.52 (158)		-	13.22 (23)	86.78 (151)		-
Waist-to-height	<0.5	57.21 (242)	42.79 (181)	<0.001	-	38.21 (115)	61.79 (186)	<0.001	-
	>0.5	21.02 (247)	78.98 (928)		5.02^*^ (3.96–6.38)	11.44 (111)	88.56 (859)		4.78^*^ (3.53–6.50)
Tobacco use	Current	39.02 (119)	60.98 (186)	<0.001	-	15.94 (40)	84.06 (211)	<0.001	-
	Former	23.56 (119)	76.44 (386)		2.08^*^ (1.52–2.83)	10.76 (61)	89.24 (506)		1.57^*^ (1.02–2.41)
	Never	32.11 (253)	67.89 (535)		1.37^*^ (1.04–1,8)	16.09 (125)	83.91 (652)		0.99 (0.66–1.45)
Alcohol use	Yes	30.14 (148)	69.86 (343)	0.78	0.96 (0.76–1.2)	15.80 (161)	84.20 (858)	0.012	0.66^*^ (0.47–0.9)
	No	30.98 (343)	69.02 (764)		-	10.98 (58)	89.02 (470)		-

* Indicates statistically significant ORs (*p* < 0.05).

HT was more prevalent in men than in women (80.1% vs. 63.3%). Male sex increased the chance of HT over 2-fold [OR 2.31; CI 1.82–2.96]. At the baseline, there was no statistically significant difference in HT prevalence between urban and rural inhabitants. Level of education was inversely associated with HT. Nearly 81.7% of participants with primary education was hypertensive in comparison with 62.5% of participants with higher education. Higher education was significantly associated with a lower chance of HT [OR 0.37, CI 0.25–0.54]. Widowhood increased the chance of HT over 1.5-fold [OR 1.67, CI 1.15–2.48] in comparison with being currently married.

The chance of HT increased along with the increase in BMI. Being overweight increased the chance of HT almost 3-fold [OR 2.93, CI 2.29–3.77], whereas obesity increased the chance of HT 8.6-fold [OR 8.57; CI 6.20–12.0] in comparison with normal body weight. A participant without abdominal obesity according to WHR also had a significantly lower chance of HT [OR 0.34; CI 0.27–0.42]. Waist-to-height ratio higher than 0.5 increased the chance of HT 5-fold [OR 5.02; CI 3.96–6.38]. Surprisingly, former smokers and never smokers had a higher chance of HT in comparison with current smokers. There was no significant difference in HT prevalence between alcohol drinkers and non-drinkers.

### 3.2. Prevalence of HT in 9-year follow-up

In the article, 9-year follow-up characteristics of the population are presented in Table 1. In the 9-year follow-up, HT was present in 85.9% of the study population. The occurrence of HT was significantly associated with age, sex, place of residence, level of education, marital status, BMI, WHR, waist-to-height ratio, and attitude toward tobacco smoking and alcohol use. Similarly as at the baseline, in the 9-year follow-up, the prevalence of HT was significantly associated with age. The chance of HT was 8.6-fold higher in the oldest vs. the youngest age group [OR 8.55; CI 4.47–16.1]. The male sex increased the chance of HT even more evidently than in the baseline [OR 3.23; CI 2.26–4.73]. In the 9-year follow-up, contrary to the baseline, the place of residence was a significant factor differentiating the prevalence of HT. The higher the level of education, the lower the chance of HT. Similarly to baseline, being widowed significantly increased the chance of HT in comparison with being currently married [OR 1.91; CI 1.12–3.52]. Additionally, in 9-year follow-up, being divorced significantly lowered the chance of HT [OR 0.61; CI 0.39–1.0].

Similarly to the baseline, obesity, according to BMI, increased the chance of HT 8-fold [OR 8.01; CI 5.20–12.8] in comparison with normal body weight. HT was significantly more prevalent in participants with abdominal obesity ascertained on the basis of WHR. In 9-year follow-up, the chance of HT was 1.6-fold higher in the former vs. current smokers [OR 1.57; CI 1.02–2.41]. Contrary to the baseline, where alcohol use was an insignificant factor associated with HT prevalence, in 9-year follow-up, current alcohol use was associated with a lower chance of HT [OR 0.66; CI 0.47–0.9].

### 3.3. Analysis of controlled HT in 9-year follow-up

We have performed the analysis of control of HT in participants with HT diagnosed at the baseline. Factors associated with control of HT are presented in Table 2. Factors that significantly differentiated the control of HT in 9-year follow-up were sex, education, BMI, and use of alcohol and BP medication.

**Table 2:** Analysis of chance for controlled hypertension after 9 years with corresponding factors.

**Characteristic**	**OR*^a^***	**95% CI*^a^***	***p*** **-value**
**Sex**			
Women	—	—	
Men	0.68	0.50, 0.92	0.014^*^
**Age**			
<45	—	—	
45–64	1.01	0.50, 2.03	0.98
>64	0.57	0.26, 1.21	0.14
**Residence**			
Rural	—	—	
Urban	0.89	0.61, 1.29	0.53
**Education**			
Primary	—	—	
Vocational School	1.71	0.91, 3.21	0.093
Secondary	2.34	1.33, 4.11	0.003^*^
College/University	2.35	1.27, 4.34	0.006^*^
**BMI**			
Normal weight	—	—	
Overweight	0.71	0.48, 1.05	0.084
Obese	0.54	0.35, 0.83	0.005^*^
**WHR**			
Normal	—	—	
Abdominal obesity	1.01	0.71, 1.44	0.96
**Tobacco use**			
Currently uses tobacco products	—	—	
Formerly used tobacco products	0.86	0.55, 1.33	0.49
Never used tobacco products	0.9	0.59, 1.38	0.64
**Alcohol use**			
No	—	—	
Yes	0.71	0.52, 0.97	0.029^*^
**BP Treatment**			
No	—	—	
Yes	3.12	2.23, 4.36	< 0.001^*^

a OR = odds ratio; CI = Confidence Interval;

* indicates statistically significant results *p* < 0.05.

Male sex decreased the chance of controlled HT after 9 years [OR 0.68; CI 0.50–0.92]. There was no statistically significant association between controlled HT and age or place of residence. On the contrary, the chance of controlled HT increased significantly with an increasing level of education. Higher and secondary education increased the chance of controlled HT over 2-fold in comparison with primary education [OR 2.35; CI 1.27–4.34, OR 2.34; CI 1.33–4.11].

Obesity significantly decreased the chance of controlled HT after 9 years in comparison with normal body weight [OR 0.54; CI 0.35–0.83]. In this model, there was no statistical significance in the association between WHR and controlled HT. Alcohol use significantly decreased the chance of controlled HT [OR 0.71; CI 0.52–0.97].

### 3.4. ROC analyses of predicted HT in 9-year follow-up

**Figure 1:**
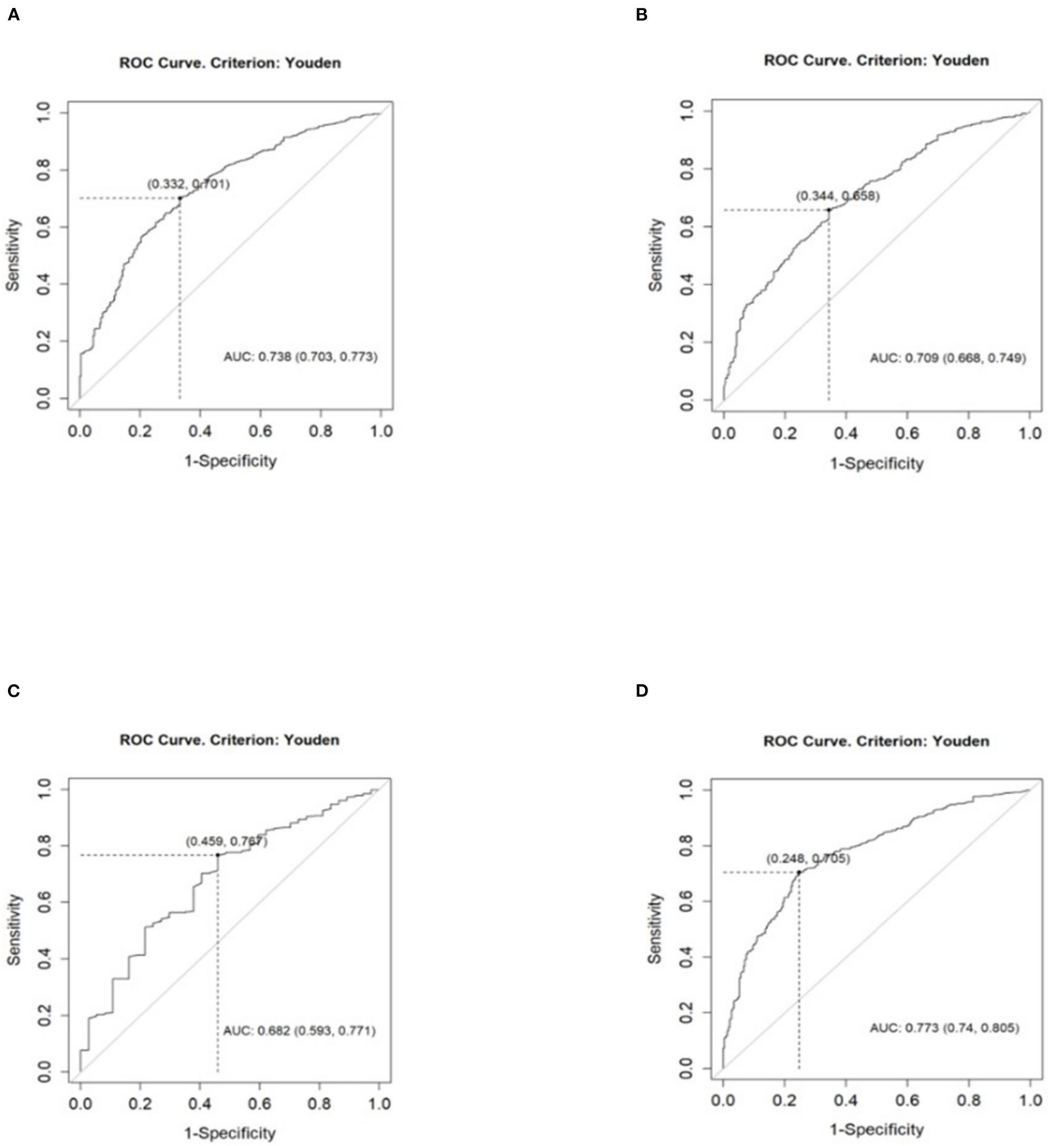
Analysis of cutoff point of anthropometric indices for higher risk of hypertension in 9-year follow-up with the use of the ROC curve. **(A)** BMI cutoff point: 26.11 kg/m2. **(B)** WHR in women cutoff point: 0.83. **(C)** WHR in men cutoff point: 0.91. **(D)** Waist-to-height ratio cutoff point: 0.53.

We performed the ROC analyses, indicating the thresholds of BMI, WHR, and waist-to-height ratio above which the risk of HT after 9 years was significantly higher. All ROC analyses are presented in Figure 1.

The ROC analysis indicated the BMI cutoff point of 26.11 kg/m2, above which the risk of HT was significantly higher [AUC 0.738 (0.703, 0.773); sensitivity 0.70; specificity 0.67; PPV 0.91; NPV 0.33]. The cutoff point of WHR in women was 0.83 [AUC 0.709 (0.668, 0.749); sensitivity 0.66; specificity 0.66; PPV 0.87; NPV 0.36]. The cutoff point of WHR in men was 0.91 [AUC 0.682 (0.593, 0.771); sensitivity 0.77; specificity 0.54; PPV 0.95; NPV 0.18]. Analyzed cutoff points correspond to current criteria of abdominal obesity according to WHR. The cutoff point of waist-to-height ratio was 0.53 [AUC 0.773 (0.74, 0.805); sensitivity 0.71; specificity 0.75; PPV 0.93; NPV 0.36].

## 4. Discussion

In this article, we analyzed the prevalence and control of HT in 9 years of observation of the Polish cohort participating in the PURE study. The prevalence of HT was significantly associated with sex, age, place of residence, level of education, marital status, BMI, WHR, waist-to-height ratio, alcohol use, and attitudes toward tobacco smoking. We found that the control of HT in 9-year follow-up was significantly lower in men, participants currently drinking alcohol, participants with a lower level of education, and elevated BMI.

The worldwide prevalence of HT is high but is not always followed by adequate BP control (15, 16). Although in the late 1990's and early 2000's, there has been an improvement in global treatment and control of HT, the control rates have plateaued in the last decade at lower levels than expected (17). The cross-sectional analysis of epidemiological data obtained in WOBASZ I (2003–2005) and WOBASZ II (2013–2014) indicates that the prevalence of HT in Poland increased by 12% in that time (11). The prevalence of HT in our cohort increased by over 16% during 9 years of observation. The prevalence of HT in our cohort is high, which is partially explained by a higher average age than in the general Polish population. On the contrary, it has been previously reported that obesity, abdominal obesity, and HT are more prevalent in Poland than in North-Western European countries (18). Central and Eastern European countries, including Poland, are characterized by a higher prevalence of raised BP, especially in men (19). In comparison with other cohorts from countries of similar socio-economic status in the PURE study, Poland was characterized by a higher average systolic blood pressure and higher prevalence of HT (4).

In our cohort, the risk of HT was 2-fold higher at the baseline and 3-fold higher at 9-year follow-up in men than in women. A higher prevalence of HT in men than women is a widely observed phenomenon in most regions of the world (19). Interestingly, the difference in HT prevalence between men and women tends to be larger in high-income countries and in Central/Eastern Europe (19). On the contrary, the increment in HT prevalence in our cohort was higher in women than in men, which has been previously observed in US and UK cohort studies (20, 21).

We have analyzed factors associated with controlled hypertension (normal blood pressure in 9-year follow-up among previously hypertensive participants). In our cohort, men were less likely to control the HT after 9 years. This observation is similar to cross-sectional analysis of WOBASZ Senior and WOBASZ II studies, where women's awareness of HT and HT control and treatment was higher than that of men (12). In the same study, despite some improvement in BP control over 7 years of observations, 70% of older adult population had poorly controlled HT. In the analysis of the global PURE study data, women were also characterized by greater awareness of HT, higher adherence to treatment, and better BP control than men (4).

The chance for controlled HT in our study was also significantly lower with excessive body weight (especially in the case of obesity) and alcohol drinking. HT control in Poland among outpatients has been recently reported as unsatisfactory (22). The majority of patients with uncontrolled hypertension (>70%) were characterized by dyslipidemia and abdominal obesity (22). In the previously mentioned WOBASZ Senior/WOBASZ II analysis, it has been also observed that the parameters of physical activity, obesity, and dyslipidemia deteriorated between studies, which may furtherly contribute to poor HT control (12). In a study by Prejbisz et al. (23), elements of metabolic syndrome, especially obesity and dyslipidemia, were significantly more prevalent among patients with resistant HT.

Obesity is a well-established contributor to HT (24). Obesity influences the risk of HT, i.e., through activation of the renin–angiotensin–aldosterone (RAS) system, activation of mineralocorticoid receptors, and activation of the sympathetic nervous system (SNS) (24). Our analyses showed that BMI above 26 kg/m2, characteristic of being overweight, and abdominal obesity significantly increased the risk of HT. Excessive body weight remains one of the biggest challenges for public health worldwide as its prevalence increased significantly over the years. According to the National Health and Nutrition Examination Survey (NHANES), the prevalence of obesity in the USA between 2000 and 2018 increased in men from 27.5% to 43.0% and in women from 33.4% to 41.9% (25). Moreover, a significant increase in obesity prevalence has been observed in the population of children and adolescents (25). In the similar time period, the analysis of controlled HT in the USA performed by Muntner et al. (26) revealed that HT control increased between 1999 and 2008, then remained stable, and then significantly decreased from 48.5% in 2013/2014 to 43.7% in 2017/2018. The study concomitantly revealed the increase in HT prevalence between the study periods (26, 27).

In our cohort, the chance for controlled HT was over 2-fold higher in participants with secondary/university education vs. primary education. The link between socio-economic status and hypertension has been previously investigated, pointing toward better awareness, treatment, and control among higher-income households (28). In accordance with our results, the higher prevalence of HT and poorer HT control in participants with a lower level of education have been observed in the HAPPIE study (16). In the analysis of the global PURE study data, a higher level of education has been significantly associated with greater awareness of HT and treatment in men, but not in women, and with greater control regardless of sex (4).

Although the prevalence of tobacco smoking has significantly decreased in Poland since the 1980's (29), the consumption of alcohol is alarmingly high. Alcohol consumption, measured in liters of pure alcohol consumed per capita, has significantly increased in the last 20 years in Poland (30), causing an alarming increase in alcohol-related deaths (31). In our cohort, alcohol consumption was associated with a higher chance of uncontrolled HT. Similarly, a higher prevalence of HT and worse HT control have been also observed in the HAPPIE study (16). Recent studies indicate that even moderate consumption of alcohol can be associated with increased BP, especially in patients with a higher cardiovascular risk and diabetes (32). Alcohol consumption of up to 1–2 drinks per day has been significantly associated with an increased risk of HT in men, whereas consumption higher than two drinks per day was associated with a higher risk of HT regardless of sex (33). In our study, we assessed only the attitudes toward alcohol drinking, without taking into consideration the amount nor type of consumed alcohol.

A recently published study indicated that the lifetime risk of developing HT is as high as 75% (34). Since elevated BP has been observed also in the young population, the importance of primary prevention and early education about HT is strongly emphasized (34). Another factor pointing toward the need for early prevention is the higher cardiovascular risk in HT patients controlled with medication in comparison with people without HT (35). One of the largest screening campaigns conducted in Poland (May Measurement Month), indicated that one-third of participants were hypertensive and half of the participants with previously diagnosed HT and on BP medication were uncontrolled (36).

Our study has some limitations to discuss. First, we present the results from a cohort study, the population of which differs slightly from the overall Polish population (by the overrepresentation of women, urban residents, and participants with higher education). Having said that, to the best of our knowledge, this is one of the very few longitudinal cohort studies conducted with a consistent protocol for over a decade in Poland. Another strength is implementing repeated BP measurements conducted by a trained professional and not relying only on self-reported data.

## 5. Conclusion

Factors significantly increasing the chance for controlled hypertension after 9 years were female sex, secondary and tertiary education, normal body weight, and avoiding alcohol drinking. In addition to proper pharmacotherapy, changes in lifestyle, with special emphasis on maintaining normal body weight, should be the basis of the prevention and control of HT.

## Data availability statement

The original contributions presented in the study are included in the article/supplementary material, further inquiries can be directed to the corresponding author: katarzyna.zatonska@umw.edu.pl.

## Ethics statement

The studies involving human participants were reviewed and approved by Positive opinion of The Bioethics Committee of the Wrocław Medical University nr KB-443/2006. The patients/participants provided their written informed consent to participate in this study.

## Author contributions

KZ made contributions to the conception and design of the article, drafting the article, and revising it. AB-R made contribution to interpretation of data and drafting the article. KP-Z made contribution to analysis and interpretation of data and revision of the manuscript. DG-D made contributions to data analysis and drafting of the manuscript. MW made contribution to data acquisition. MK made contribution to data analysis and presentation of the results. AS made contributions to the conception and design of the article and revising the manuscript. All authors provided approval for publication of the content and agree to be accountable for all aspects of the article in ensuring that questions related to the accuracy or integrity of any part of the article are appropriately investigated and resolved.
